# Hyperthyroidism Due to Graves Disease After Radiofrequency Ablation

**DOI:** 10.1210/jcemcr/luad056

**Published:** 2023-06-08

**Authors:** Elizabeth A McAninch, Kaniksha Desai, Karen C McCowen, Lisa A Orloff

**Affiliations:** Division of Endocrinology, Gerontology and Metabolism, Stanford University Medical Center, Stanford, CA 94305, USA; Division of Endocrinology, Gerontology and Metabolism, Stanford University Medical Center, Stanford, CA 94305, USA; Division of Endocrinology, University of California San Diego, San Diego, CA 92093, USA; Department of Otolaryngology-Head and Neck Surgery, Stanford University Medical Center, Stanford, CA 94305, USA

**Keywords:** multinodular goiter, hyperthyroidism, thyroid autoimmunity, thyroid nodule, radiofrequency ablation, Graves disease

## Abstract

Management options for benign, autonomously functioning, and malignant thyroid nodules were limited to surgery or targeting by radioactive iodine before the availability of radiofrequency ablation (RFA). Despite being a relatively new technique, RFA may be favored for patients of high surgical risk, and for those who wish to avoid hypothyroidism. Although insurance coverage for the procedure can be a significant barrier, several groups of investigators have shown improved quality of life for RFA compared to surgery, due to the less invasive nature and favorable risk profile. Hyperthyroidism due to transient thyroiditis is a known risk of RFA, secondary to direct trauma and subsequent thyroid hormone release. Here we present a case of an adult with large, symptomatic, multinodular goiter, with no prior history of thyroid autoimmunity, who underwent RFA with successful volume reduction of two nodules, but who developed acute hyperthyroidism due to Graves disease eight weeks after RFA. Larger studies evaluating the risks of RFA should evaluate for incident hyperthyroidism, specifically for Graves disease/thyroid autoimmunity, as this could represent an additional risk of the procedure.

## Introduction

Radiofrequency ablation (RFA) is an increasingly popular, less invasive treatment option for malignant, autonomously functioning, and benign, symptomatic thyroid nodules [1]. Before the availability of RFA, management options for such thyroid neoplasia would have been limited to more invasive surgical approaches or targeting by radioactive iodine. Even if unilateral lobectomy is pursued, there is 10% to 40% [2] risk of developing hypothyroidism, requiring lifelong thyroid hormone replacement therapy and monitoring. Although insurance coverage can be a significant barrier in the United States, some patients may prefer RFA over surgery because of the relative risk profile and suggestion of improved quality of life [1, 3-5]. Overt or subclinical thyrotoxicosis due to transient thyroiditis is known to occur in a minority of cases [6-8], thought to be due to direct trauma and subsequent thyroid hormone release.

Here we present a case of an adult with large, symptomatic multinodular goiter who underwent RFA with successful volume reduction of two nodules, but who developed acute hyperthyroidism due to Graves disease (GD), eight weeks after RFA.

## Case Presentation

In 2009, a 32-year-old male with no past medical history presented for routine physical examination with his primary care physician. He was born and raised in the United States. He had no family history of functional or structural thyroid disease. He denied a history of head and neck irradiation. He had never smoked. Enlarged thyroid was palpated on exam, and thus he was referred for ultrasound. At that time, his right lobe measured 6 × 3 cm, and left lobe 6.5 × 3.5 cm. Both lobes were almost completely occupied by innumerable complex nodules. There was normal blood flow within the thyroid parenchyma. Corresponding thyroid function tests from that time were not available. Fine needle aspiration of the dominant right nodule was benign (size undescribed).

## Diagnostic Assessment

**Figure 1.**
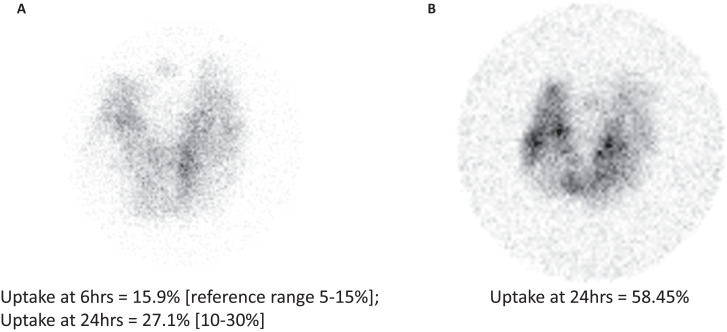
123-I thyroid uptake and scan results. (A) Obtained almost 3 years prior to radiofrequency ablation (TSH 0.30 uIU/mL [2.08 pmol/L]), and (B) 3 months after radiofrequency ablation of right and isthmic thyroid nodules (TSH <0.01 uIU/mL [<0.07 pmol/L]).

Endocrinology was consulted in 2017. At that time, he denied compressive symptoms. On exam he had had a negative Pemberton's sign. On laboratory workup, his serum TSH was 0.38 uIU/mL [2.64 pmol/L] (0.27–4.20 uIU/mL; 1.88–29.2 pmol/L) and free T4 was 1.22 ng/dL [0.04 nmol/L] (0.76–1.46 ng/dL; 0.03–0.05 ng/dL). He underwent a repeat thyroid ultrasound which showed a right lobe that was 7.4 cm in long axis and left lobe that was 8.8 cm in long axis. The gland was completely replaced by nodularity and the report described at least 6 different thyroid nodules in the right, isthmus, and left lobes. In 2018, he remained asymptomatic, and on laboratory workup his serum thyrotropin (TSH) was 0.30 uIU/mL (2.08 pmol/L) with free thyroxine (T4) of 1.18 ng/dL (0.04 nmol/L). One year later, ultrasound showed right lobe dimensions at 8.0 × 5.2 × 4.2 cm and left lobe at 8.1 × 4.0 × 3.5 cm. All 6 previously noted nodules were present and had grown; specifically, a right nodule was noted to now extend below the clavicle. In 2019 he underwent an iodine 123 (123-I) uptake and scan, due to persistently low-normal serum TSH, in which his thyroid was noted to be enlarged (right and left long axes 8 cm). The 123-I uptake at 6 hours was 15.9% (reference range, 5%–15%) and at 24 hours 27.1% (10%–30%), and showed a heterogeneous and patchy pattern, with the highest uptake in the medial left lobe, and decreased uptake in the right mid-inferior area, possibly suggesting a right-sided cold nodule (Fig. 1A). The technically normal serum TSH and normal 24-hour uptake were together interpreted as representing functional endogenous euthyroidism, and suggested lack of prevalent GD. At that time, Nuclear Medicine offered to administer 29 mCi 131-I for his growing, multinodular goiter, but the patient declined. At follow-up with his endocrinologist in 2021, he had become mildly symptomatic, with increased throat clearing and discomfort with swallowing. His serum TSH continued to be at the lower end of normal, at 0.32 uIU/mL (2.22 pmol/L) with a normal free T4 of 1.23 ng/dL (0.04 nmol/L). Given that he was now symptomatic, he was counseled on treatment options for his enlarging thyroid nodules, including surgery and radioactive iodine, but patient declined these options and requested referral to an institution that offered radiofrequency ablation (RFA).

**Figure 2.**
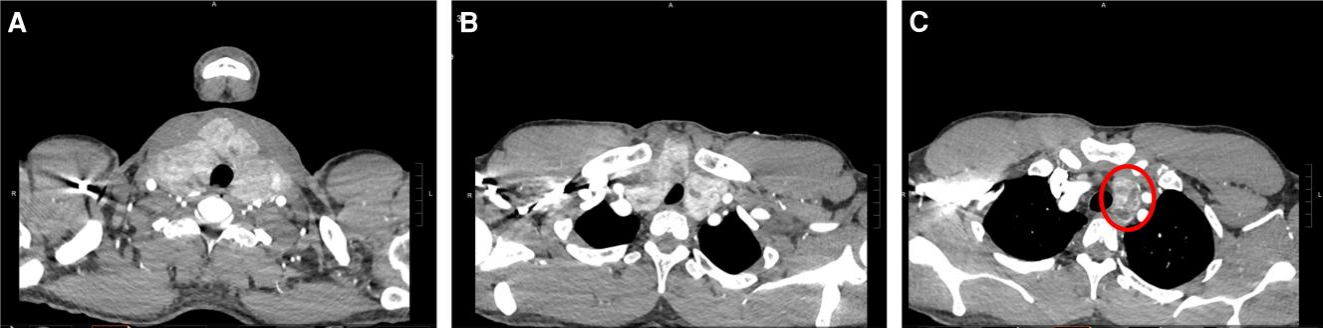
Computed tomography angiogram of the chest performed 4 months prior to radiofrequency ablation, when the patient presented with COVID-associated deep vein thrombosis. In the most superior axial image, the goiter is evident (A) and displacing the trachea posteriorly; (B) thyroid extends below the clavicles with tracheal narrowing; (C) Substernal extension from the left thyroid [circle].

In August 2021, now age 44, he presented to Stanford University Medical Center with acute COVID-19 and a left lower extremity deep vein thrombosis. Due to concomitant shortness of breath, he underwent a computed tomography angiogram of the chest (Fig. 2). The study was negative for pulmonary embolism but was significant for massive infraclavicular (Fig. 2B) and substernal thyroid goiter (Fig. 2C), with tracheal deviation.

**Figure 3.**
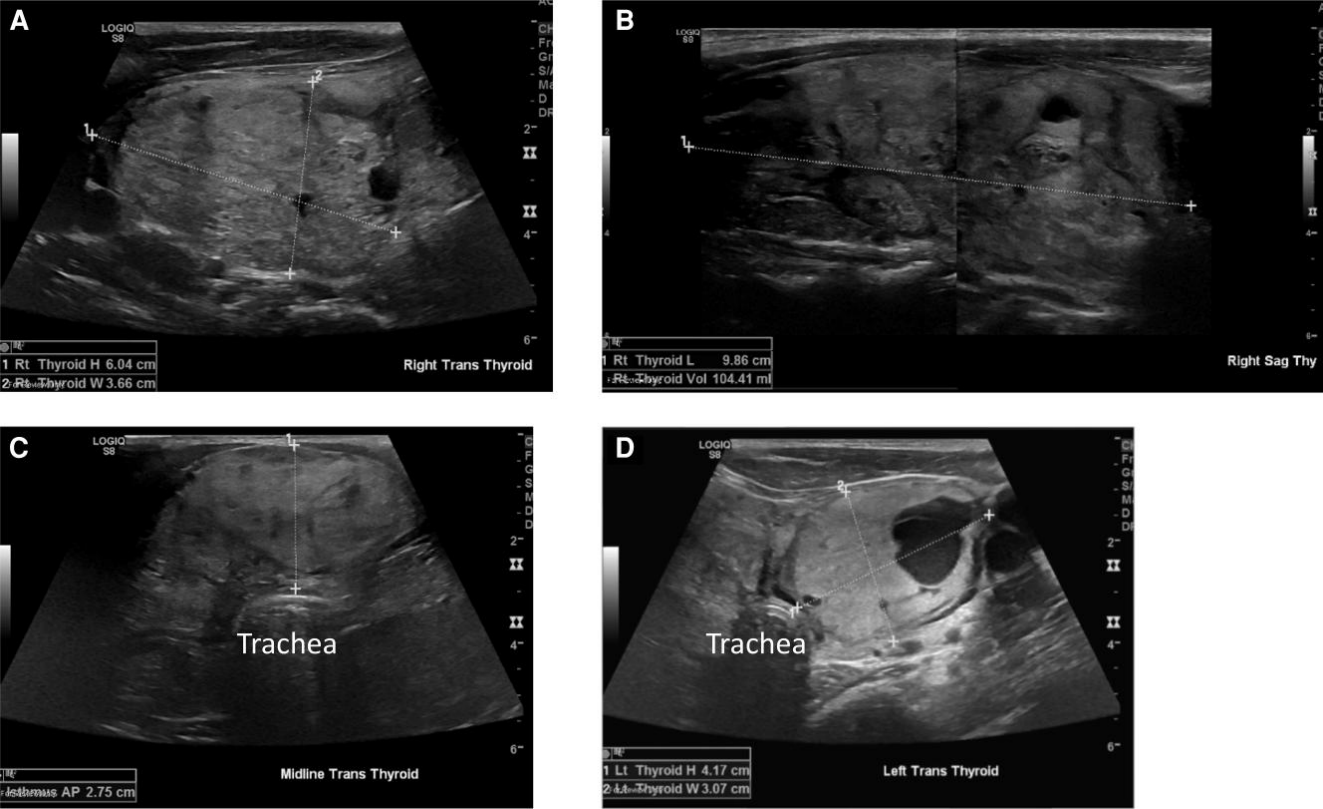
Thyroid ultrasound images 1 month before radiofrequency ablation of right and isthmic nodules. Right nodule in transverse (A) and sagittal (B) views; (C) isthmus nodule; left nodule (D).

He consulted with Endocrine Head & Neck Surgery in November 2021, during which he complained of exertional dyspnea, and feeling that his “windpipe [was] compressed.” Bedside ultrasound at that time (Fig. 3) was notable for large, multinodular goiter extending superiorly to approximate the submandibular glands bilaterally, and with substernal extension obscuring the inferior borders. In addition, the trachea was noted to be compressed from 3 sides and deviated posteriorly. There were no suspicious lymph nodes in the central or lateral neck. It was recommended that the patient undergo total thyroidectomy for growing, now symptomatic, goiter, which he again declined. He wished to try RFA as a less invasive approach to try to shrink his nodules. Before RFA, repeat fine needle aspirations of his dominant right and left thyroid nodules were both benign on cytology. His serum TSH was 0.29 uIU/mL (2.01 pmol/L) (reference range, 0.27–4.20 uIU/mL; 1.88–29.2 pmol/L), and his free T4 was 1.44 ng/dL (0.05 nmol/L) (reference range, 0.93–1.70 ng/dL; 0.03–0.06 nmol/L). Per research protocol, baseline testing included anti-thyroid peroxidase and anti-thyroglobulin antibodies (both negative), and serum thyroglobulin was 1442.9 ng/mL (5002.8 nmol/L) (reference, <50.0 ng/mL; <173 ng/mL). He was counseled on the risks and benefits of all treatment options and wished to pursue RFA.

## Treatment

In December 2021, ultrasound-guided RFA of the right-sided and isthmic nodules was performed using a trans-isthmic, moving-shot technique, at a setting of 50 to 70 watts, for a total of approximately 32:39 minutes (run-time). A total of 109 448 watts were delivered. The plan was to pursue RFA of left nodule in the future.

## Outcome and Follow-up

**Figure 4.**
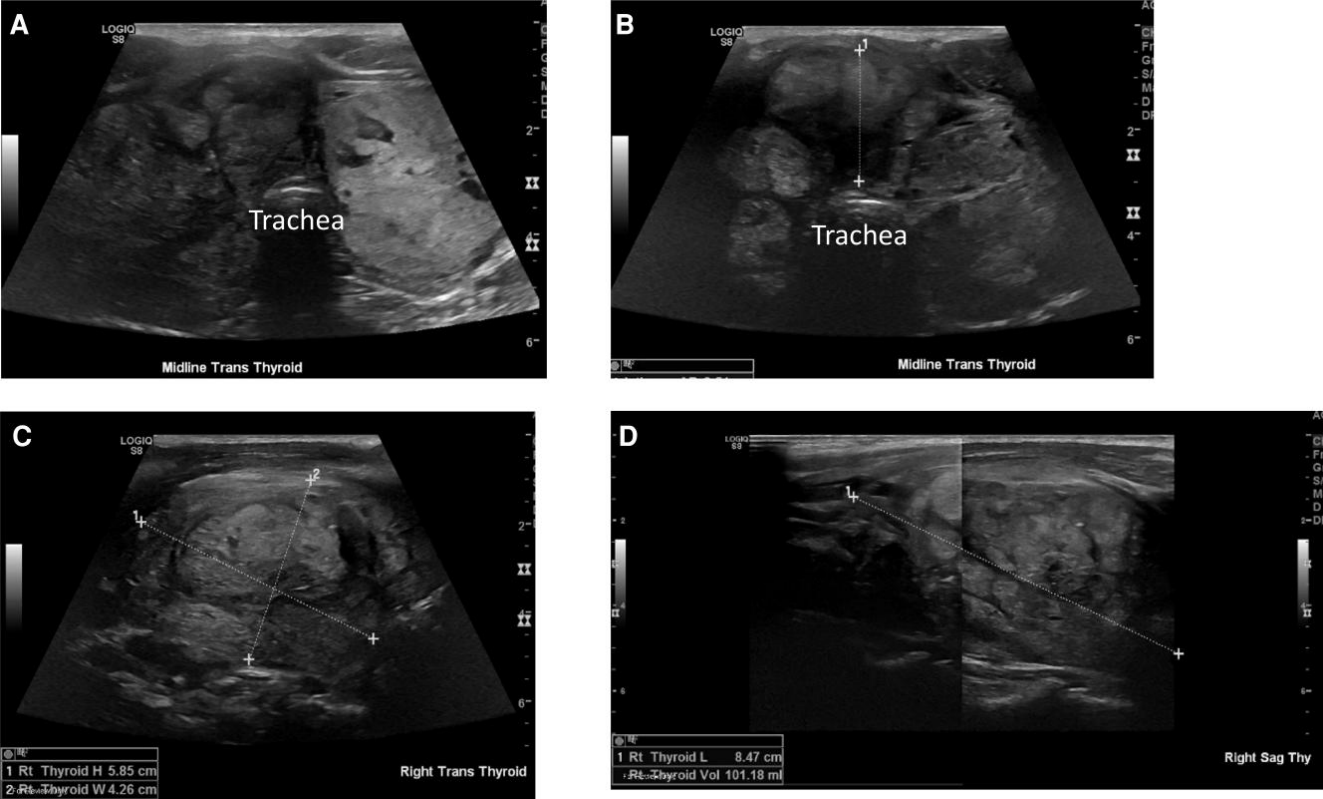
Thyroid ultrasound images 8 weeks after radiofrequency ablation of right and isthmic nodules. (A) Thyroid in transverse plane; (B) isthmus nodule; (C) right nodule in transverse and sagittal (D) views.

In February 2022, at his 8-week follow-up, the patient reported that his mild neck compressive symptoms and procedural soreness had resolved, but that he had been experiencing increased anxiety, insomnia, and elevated heart rate for about 2 weeks. On physical examination, he was afebrile, with regular heart rate, and had no overt proptosis. Bedside ultrasound showed that his right nodule volume had reduced by 37% (Fig. 4). Biochemical evaluation elucidated primary hyperthyroidism: serum TSH <0.01 uIU/mL (<0.07 pmol/L), free T4 4.71 ng/dL (0.163 nmol/L), free triiodothyronine (T3) 14.5 pg/mL (3.19 pmol/L) (reference range, 2.0–4.4 pg/mL; 0.44–0.97 pmol/L), and positive thyroid-stimulating immunoglobulin (TSI) 4.4 (≤1.3). He was then referred to Stanford Endocrinology for new-onset hyperthyroidism. He was unvaccinated for COVID-19 and denied known interval COVID-19 reinfection. He denied recent social/physical stressors.

He had repeat 123-I uptake and scan (Fig. 1B) showing multinodular appearance, diffusely heterogeneous distribution of the radiotracer in both lobes, without distinct hyper- or hypofunctioning nodules. An area of photopenia in the inferior right thyroid lobe was thought to correspond to recently radiofrequency-ablated nodule. The uptake at 24 hours was 58.45% (normal, 10%-30%), consistent with GD. He was counseled on his diagnosis and treatment options. Again, it was felt that, due to the size of his goiter, the best treatment option would be surgery; he declined all treatment options for GD. Two months after 123-I uptake and scan, his laboratory tests were repeated: serum TSH <0.01 uIU/mL (<0.07 pmol/L), free T4 2.59 ng/dL (0.09 nmol/L), total T3 219 ng/dL (7.59 nmol/L) (80–200 ng/dL; 2.77–6.93 nmol/L), TSI continued to be positive at 1.9, and anti-thyroid peroxidase antibody was negative. A few months later we received a message that he had moved out of state and requested a referral to a local surgeon.

## Discussion

RFA under ultrasound guidance is a nonsurgical option for the management of select thyroid nodules and reduces thyroid nodule volume. RFA has the advantage of preserving non-nodule thyroid parenchyma, thereby preserving thyroid function and avoiding the need for lifelong thyroid hormone supplementation or replacement in contrast to lobectomy or total thyroidectomy [1]. RFA also avoids a surgical scar and surgical recovery, and the complication rate is equal to or lower than that of surgery (including rates of recurrent laryngeal nerve injury and hypoparathyroidism) [4, 5]. Indications for thyroid nodule RFA include compressive symptoms, autonomous function, progressive enlargement, and cosmetic deformity for nodules at least 2 cm in diameter and in an anatomically favorable, accessible location [1]. Risks of RFA include, but are not limited to, unilateral or bilateral laryngeal nerve injury, other nerve injury, skin burn, nodule rupture, hypoparathyroidism, hypothyroidism requiring lifelong thyroid hormone supplementation, hyperthyroidism [6], bleeding, infection, and scarring, and incomplete nodule regression with possible need for further RFA or even surgery [7].

This gentleman with longstanding, symptomatic, substernal, multinodular goiter repeatedly declined thyroidectomy and opted for RFA. He then developed acute hyperthyroidism after RFA of 2 of his large nodules, and subsequent workup suggested the etiology to be most consistent with GD. Success of RFA is determined by subjective symptom improvement and volume reduction; he had good volume reduction but developed hyperthyroidism.

To our knowledge, this is the first reported case of hyperthyroidism due to GD developing acutely after RFA. Incident GD after percutaneous ethanol ablation has been described [9], suggesting that there may be a mechanistic connection between thyroid trauma, thyroid antigen release, and generation of TSI or thyrotropin-receptor antibodies (TRAb). Likewise, it has also been reported that patients with thyroiditis (both autoimmune and granulomatous) can have positive TRAb [10]. In the patients with thyroiditis, no induction of endogenous hyperthyroidism was noted [10], but this could be due to the diffuse damage inherent in thyroiditis as compared to the focal damage related to RFA in our case.

There are several limitations to determining causality in this case, one being the lack of preprocedural TSI or TRAb. Thus, it remains possible that he had a subclinical/smoldering GD prior to RFA [9]. However, prior to RFA, he was euthyroid, had no evidence of thyroid autoimmunity, had not been exposed to iodinated contrast for over 4 months, and in 2018 his 24-hour 123-I uptake was normal; thus, the overall preprocedural clinical picture was not likely consistent with GD.

There are possible adverse effects of RFA that are unknown or under-recognized. As RFA gains in popularity and utilization, more studies should quantify the risks of hyperthyroidism, and when detected, evaluate for GD. In this case, and the other reported after percutaneous ethanol ablation [9], it would have been interesting to know the preprocedural TSI/TRAb values, so investigators should consider obtaining this if deemed cost effective.

## Learning Points

- RFA can effectively result in volume reduction of symptomatic thyroid nodules.
- RFA may offer improved quality of life compared with more invasive surgical approaches.
- RFA can cause hyperthyroidism, which has been attributed to thyroiditis in the majority of previously reported cases.
- This patient developed hyperthyroidism due to Graves disease developing acutely after RFA. Incident Graves disease after percutaneous ethanol ablation has been described, suggesting a possible mechanistic connection between thyroid trauma, thyroid antigen release, and triggered or exacerbated thyroid autoimmunity.

## Contributors

All authors made individual contributions to authorship. E.A.M., K.M., and L.A.O. were involved in the diagnosis and management of this patient. E.A.M. drafted the manuscript. L.A.O. was responsible for the patient's RFA procedure. K.D., K.M., and L.A.O. revised the manuscript. All authors reviewed and approved the final draft.

## Data Availability

Data sharing is not applicable to this article as no datasets were generated or analyzed during the current study.

## References

[1] Orloff LA, Noel JE, Stack BC, Jr., et al Radiofrequency ablation and related ultrasound-guided ablation technologies for treatment of benign and malignant thyroid disease: an international multidisciplinary consensus statement of the American Head and Neck Society Endocrine Surgery Section with the Asia Pacific Society of Thyroid Surgery, Associazione Medici Endocrinologi, British Association of Endocrine and Thyroid Surgeons, European Thyroid Association, Italian Society of Endocrine Surgery Units, Korean Society of Thyroid Radiology, Latin American Thyroid Society, and Thyroid Nodules Therapies Association. Head Neck. 2022;44(3):633‐660. 34939714 10.1002/hed.26960

[2] Dou Y, Chen Y, Hu D, Su X. The recovery of thyroid function in low-risk papillary thyroid cancer after lobectomy: a 3-year follow-up study. Front Endocrinol (Lausanne). 2020;11:619841. 33633689 10.3389/fendo.2020.619841 PMC7899978

[3] Yue WW, Wang SR, Li XL, et al Quality of life and cost-effectiveness of radiofrequency ablation versus open surgery for benign thyroid nodules: a retrospective cohort study. Sci Rep. 2016;6(1):37838. 27883069 10.1038/srep37838 PMC5121639

[4] Che Y, Jin S, Shi C, et al Treatment of benign thyroid nodules: comparison of surgery with radiofrequency ablation. AJNR Am J Neuroradiol. 2015;36(7):1321‐1325. 25814656 10.3174/ajnr.A4276 PMC7965284

[5] Bernardi S, Dobrinja C, Carere A, et al Patient satisfaction after thyroid RFA versus surgery for benign thyroid nodules: a telephone survey. Int J Hyperthermia. 2018;35(1):150‐158. 30107758 10.1080/02656736.2018.1487590

[6] Wang N, Zheng B, Wu T, et al Thyroid dysfunction following radiofrequency ablation for benign thyroid nodules: more likely to occur within one-week and in high-risk population. Int J Hyperthermia. 2021;38(1):1060‐1068. 34265235 10.1080/02656736.2021.1950849

[7] Lim JY, Kuo JH. Thyroid nodule radiofrequency ablation: complications and clinical follow up. Tech Vasc Interv Radiol. 2022;25(2):100824. 35551808 10.1016/j.tvir.2022.100824

[8] Muhammad H, Santhanam P, Russell JO. Radiofrequency ablation and thyroid nodules: updated systematic review. Endocrine. 2021;72(3):619‐632. 33449296 10.1007/s12020-020-02598-6

[9] Regalbuto C, Le Moli R, Muscia V, Russo M, Vigneri R, Pezzino V. Severe Graves’ ophthalmopathy after percutaneous ethanol injection in a nontoxic thyroid nodule. Thyroid. 2012;22(2):210‐213. 22196093 10.1089/thy.2011.0315

[10] Angell TE, Van Benschoten O, Cohen DA, Haas AV, Alexander EK, Marqusee E. Positive thyrotropin receptor antibodies in patients with transient thyrotoxicosis. Endocr Pract. 2018;24(6):512‐516. 29624097 10.4158/EP-2018-0059

